# Inhibition of TGF-β Signaling and Decreased Apoptosis in IUGR-Associated Lung Disease in Rats

**DOI:** 10.1371/journal.pone.0026371

**Published:** 2011-10-20

**Authors:** Miguel Angel Alejandre Alcázar, Rory E. Morty, Lisa Lendzian, Christina Vohlen, Iris Oestreicher, Christian Plank, Holm Schneider, Jörg Dötsch

**Affiliations:** 1 Department of Pediatrics and Adolescent Medicine, University of Cologne, Cologne, Germany; 2 Department of Internal Medicine, University of Giessen Lung Centre, Justus-Liebig University, Giessen, Germany; 3 Department of Pediatrics and Adolescent Medicine, University Hospital Erlangen, Erlangen, Germany; 4 Department of Neonatology, Charité University Medical Center, Berlin, Germany; University of Tübingen, Germany

## Abstract

Intrauterine growth restriction is associated with impaired lung function in adulthood. It is unknown whether such impairment of lung function is linked to the transforming growth factor (TGF)-β system in the lung. Therefore, we investigated the effects of IUGR on lung function, expression of extracellular matrix (ECM) components and TGF-β signaling in rats. IUGR was induced in rats by isocaloric protein restriction during gestation. Lung function was assessed with direct plethysmography at postnatal day (P) 70. Pulmonary activity of the TGF-β system was determined at P1 and P70. TGF-β signaling was blocked *in vitro* using adenovirus-delivered Smad7. At P70, respiratory airway compliance was significantly impaired after IUGR. These changes were accompanied by decreased expression of TGF-β1 at P1 and P70 and a consistently dampened phosphorylation of Smad2 and Smad3. Furthermore, the mRNA expression levels of inhibitors of TGF-β signaling (Smad7 and Smurf2) were reduced, and the expression of TGF-β-regulated ECM components (e.g. collagen I) was decreased in the lungs of IUGR animals at P1; whereas elastin and tenascin N expression was significantly upregulated. *In vitro* inhibition of TGF-β signaling in NIH/3T3, MLE 12 and endothelial cells by adenovirus-delivered Smad7 demonstrated a direct effect on the expression of ECM components. Taken together, these data demonstrate a significant impact of IUGR on lung development and function and suggest that attenuated TGF-β signaling may contribute to the pathological processes of IUGR-associated lung disease.

## Introduction

The term fetal programming reflects the assumption that a temporary environmental influence during intrauterine development may lead to permanent alterations of physiological processes later in life [1], [2], [3]. Intrauterine undernourishment can represent such an environmental factor, leading to intrauterine growth restriction (IUGR) and, in most cases, to low birth weight (LBW). Furthermore, there is evidence that being born with LBW also has an impact on lung development and function [4], [5], [6].

Organogenesis of the lung occurs in five stages: 1.) the embryonic stage, 2.) the pseudoglandular stage, 3.) the canalicular stage, 4.) the saccular stage, and 5.) the alveolar stage. A sixth stage - microvascular maturation - has also been proposed [7]. The process of lung development is highly regulated and thus susceptible to modification by perinatal environmental conditions [7], [8]. Consequently, disturbed intrauterine growth may induce changes in lung structure, which predispose lungs to later disease. Several observational studies have described decreased lung function with reduced forced expiratory volume in one second (FEV_1_) in young infants [9], in school children [10], and in young adults born with LBW [11], [12]. The terms IUGR and “small for gestational” age (SGA) are often used synonymously, but the distinction between them is important. IUGR is the pathological form of SGA. It affects growth and predisposes to diseases later in life. Intrauterine protein restriction has been shown to be a reliable animal model of IUGR [13], [14], and several animal studies addressing structural changes of the pulmonary system have also demonstrated reduced lung function following IUGR [15], [16], [17].

Lung structure and function are determined during early and late lung development [18], [19], [20]. While the pathogenic processes leading to IUGR-associated lung disease have not yet been elucidated, extracellular matrix (ECM) and its maintenance during alveolarization is thought to play a pivotal role in disease pathogenesis [21], [22]. Disruption of critical signaling pathways may be involved [19], [23], including signaling by the transforming growth factor (TGF)-β superfamily [23], [24]. TGF-β signaling is initiated by binding of TGF-β to the type II TGF-β receptor (TβRII), which then forms a complex with either the type I receptor (TβRI) or activin A receptor type II-like 1 (Acvrl1, also called ALK-1). The type I receptor transmits signals within the cell via second-messenger Smad proteins, namely Smad1-Smad4, or by Smad-independent pathways [25]. TGF-β signaling is also regulated by Smad6 and Smad7, inhibitory Smads which antagonize TGF-β signaling. Several studies have indicated that TGF-β signaling plays a critical and finely tuned role in pulmonary branching and alveolarization [18], [23], since TGF-β ligands inhibited airway branching *in vitro* [26], [27]. Furthermore; abrogation of TGF-β signaling by genetic ablation of TβRII [28], Smad2, Smad3 or Smad4 enhanced lung branching *in vitro* [29]. Consistent with these observations, overexpression of Smad7 promoted lung branching [30]. However, conditional overexpression of the TGF-β1 gene in the mouse lung during the postnatal period disrupts lung development [31]. Interestingly, Smad3 deficiency in mice results in progressive airspace enlargement with age [32]. These studies implicate TGF-β signaling as a regulator of lung branching and alveolarization. Together with reports that TGF-β might be associated with fetal growth in pregnancy [33], they have led us to hypothesize that IUGR due to protein restriction during gestation may influence TGF-β signaling and contribute to impaired lung function and structural changes in later life.

## Methods

### Induction of intrauterine growth restriction

All procedures performed on animals were done in accordance with the German regulations and legal requirements and were approved by the local government authorities (Regierung von Mittelfranken, AZ # 621-2531.31-11/02 and AZ # 621-2531.31-14/05).

Adult and neonatal Wistar rats were housed in humidity- and temperature controlled rooms on a 12∶12-h light-dark cycle. IUGR in rats was induced as previously described [14]. In brief, virgin female Wistar rats were obtained from our own colony. Dams were time-mated by the appearance of sperm plugs, then fed either a normal diet containing 17.0% protein (control group) or a low protein isocaloric diet containing 8.0% protein (casein) throughout pregnancy (IUGR group). Diets were obtained from Altromin, Germany (# C1000, C1003). Rats delivered spontaneously at day 23 of pregnancy. On the first postnatal day (P1) the litters were reduced to six pups per dam. During lactation, dams were fed standard chow. Weaning was at P23. IUGR and control animals were sacrificed at P1 and P70 and assigned to four groups: IUGR P1, Control P1, IUGR P70, and Control P70. Body weight and weight of the lung were obtained immediately after sacrificing the animals. Means ± standard error of the mean were calculated.

### Measurement of airway responsiveness

At P70, lung function was assessed by measuring respiratory system dynamic compliance (C_dyn_) with direct plethysmography (FinePointe™ RC; Wellington, NC, USA). C_dyn_ is defined as a measure of the ability of the lung to distend in response to pressure. Decreased compliance means that a greater change in pressure is needed for a given change in volume, as in atelectasis, edema, fibrosis, pneumonia, or absence of surfactant. Rats were deeply anesthetized by intramuscular injection of ketamine (100 mg/kg body weight) and midazolame (5 mg/kg body weight), tracheotomized and ventilated. C_dyn_ was measured at baseline.

### Processing of lung tissue and morphometric analysis

Following anesthesia with ketamine (100 mg/kg body weight) and midazolame (5 mg/kg body weight) the animals were exsanguinated by aortic transection. Neonatal animals at P1 were euthanized by decapitation. The right lobe of the lung was removed after ligation of the bronchus, and one portion was immediately snap-frozen in liquid nitrogen for mRNA and protein analysis. The left lobe was inflated via tracheotomy and pressure-fixed at 20 cm H_2_O with 4% (mass/vol) paraformaldehyde, and the trachea was ligated. Lungs and hearts were excised *en bloc*, submersed in 4% (mass/vol) paraformaldehyde overnight for paraffin embedding and sectioning as described previously [34]. Paraffin sections (1 µm) were mounted on poly-L-lysine-coated glass slides, dewaxed with xylene (3–5 min) and rehydrated in a graduated series of ethanol solutions (100%, 95%, and 70% (vol/vol), finally PBS). The mean linear intercept (MLI) and septal thickness were determined on sections stained for smooth muscle actin and counter-stained with hematoxylin and eosin as described previously [35], [36].

### RNA extraction and real-time PCR

Total RNA was isolated from unfixed lung tissue or cultured cells as previously described [37], followed by DNase treatment to remove any contaminating genomic DNA. Total RNA was screened for mRNA encoding ALK-1, ALK-5, TβRII, TβRIII, Smad2, Smad2, Smad3, Smad4, Smad6, Smad6, Smurf2, elastin (Eln), tenascin N (TenN), collagen I (Coll I), collagen III (Coll III), fibrillin (Fbl), matrix metalloproteinases (MMP) 2, MMP 9, tissue inhibitor of matrix metalloproteinases (TIMP) 1, TIMP 2, plasminogen activator inhibitor-1 (PAI-1), surfactant protein A (SP-A), SP-C, and SP-D. Quantitative changes in mRNA expression were assessed by quantitative real-time PCR as described previously [14] using the IQ TM SYBR-Green © Supermix and a BioRad iQ5-Cycler (Bio-Rad Laboratories, Hercules, CA, USA) or the 7500 Real-time PCR system (Applied Biosystem, Foster City, CA, USA) [14]. In all samples, the relative amount of specific mRNA was normalized to the ubiquitously expressed glyceraldehyd-3-phosphat-dehydrogenase (GAPDH) and β-actin gene. Primer pairs and TaqMan probes are listed in Table 1. Oligonucleotides were designed with Primer Express software (Perkin-Elmer, Foster City, CA, USA).

**Table 1:** Primer pairs and TaqMan probes used.

**PAI-1**	forward 5′-TCCGCCATCACCAACATTTT-3′reverse 5′-GTCAGTCATGCCCAGCTTCTC-3′probe 5′(FAM)- CCGCCTCCTCATCCTGCCTAAGTTCTCT-(TAMRA)3′
**TGFβ-1**	forward 5′-CACCCGCGTGCTAATGGT-3′reverse 5′-GGCACTGCTTCCCGAATG-3′probe 5′(FAM)-ACCGCAACAACGCAATCTATGACA-(TAMRA)3′
**tgfbr1**	forward 5′-ACCGCGTGCCAAATGAAGAGGAT-3′reverse 5′-GGTAAACCTGATCCAGACCCTGAT-3′
**Tgfbr2**	forward 5′-GAGCAACTGCAGCGTCACC-3′reverse 5′-CCAGAGTAATGTTCTTGTCGTTC-3′
**Tgfbr3**	forward 5′-CTTGACAGCAGAAACAGAGG-3′reverse 5′-AAACACTTGATCTTCTCCCAC-3′
**Smad2**	forward 5′-AATTACATCCCAGAAACACCAC-3′reverse 5′-TGTCCATACTTTGGTTCAACTG-3′
**Smad3**	forward 5′-CACCAGTGCTACCTCCAGTGT-3′reverse 5′-TAGTGTTCTCGGGGATGGAA-3′
**Smad4**	forward 5′-CTACTTACCACCATAACAGCACTACCA-3′reverse 5′-GTGCTGAAGATGGCCGTTTT-3′
**Smad6**	forward 5′-CCCCCTATTCTCGGCTGTCT-3′reverse 5′-TGGTGGCCTCGGTTTCA-3′
**Smad7**	forward 5′-AGATACCCGATGGATTTTCTCAAA-3′reverse 5′-TCGTTCCCCCGGTTTCA-3′
**Smurf2**	forward 5′-GGTCTCAGCGACATAGAAATTACATG-3′reverse 5′-TGTTGTGTTGTCCTCTGTTCATAGC-3′
**SARA**	forward 5′-GCCAACGTGCCTATCCTAATTC-3′reverse 5′-ACTGCCCTTTCCTGTTGTCTGA-3′
**Elastin**	forward 5′-GAAAACCCCCGAAGCCCT-3′reverse 5′-CCCCACCTTGATATCCCAGG-3′
**Tenascin N**	forward 5′-AGGTGGACTATTACAAGCTTCGGTAT-3′reverse 5′-GCAGACCGGTGATGTCATATCTAC-3′
**Collagen I α**	forward 5′-AGAGCGGAGAGTACTGGATCGA-3′reverse 5′-CTGACCTGTCTCCATGTTGCA-3′probe 5′(FAM)-CAAGGCTGCAACCTGGATGCCATC-(TAMRA)3′
**Collagen III**	forward 5′-GGACCTCCTGGTGCTATTG -3′reverse 5′-GAATCCAGGGATACCAGCTG-3′
**Fibrillin**	forward 5′-TGCTCTGAAAGGACCCAATGT-3′reverse 5′-CGGGACAACAGTATGCGTTATAAC-3′
**mCollagen I**	forward 5′-TCACCTACAGCACCCTTGTGG -3′reverse 5′-CCCAAGTTCCGGTGTGACTC-3′
**mTenascin N**	forward 5′-AGAAGCTGAACCGGAAGTTGAC-3′reverse 5′-CGTCTGGAGTGGCATCTGAAA-3′
**mElastin**	forward 5′-GGCTTTGGACTTTCTCCCATT-3′reverse 5′-CCACCTTGGTATCCCAGGG-3′
**mMMP-2**	forward 5′-ATGCGGAAGCCAAGATGTG-3′reverse 5′-GTCCAGGTCAGGTGTGTAAC-3′
**mβ-Actin**	forward 5′-GACATCAGGAAGGATCTCTATG-3′reverse 5′-CTTCTGCATCCTGTCAGCAA-3′
**SP-A**	forward 5′-GGGATAGTAGCCATGTCACTGTGT-3′reverse 5′-CGTCTGTCACATTGCACTTGATAC-3′
**SP-C**	forward 5′-CCTGAGTGAACACACAGACACCAT-3′reverse 5′-GTCAGGAGCCGCTGGTAGTC-3′
**SP-D**	forward 5′- TAGAGGCTGCCTTTTCTCGCTAT -3′reverse 5′-GCCGCCCTGAAGATTTTGT-3′
**TIMP-2** *	forward 5′-TCAAAGGACCTGACAAGGACATC-3′reverse 5′-CGCCTTCCCTGCAATTAGATAT-3′probe 5′(FAM)-TCTACACGGCCCCCTCCTCAGCA-(TAMRA)3′
**MMP-2** *	forward 5′-CTGGAGATACAATGAAGTAAAGAAGAAAAT-3′reverse 5′-CACGACTGCATCCAGGTTATCA-3′probe 5′(FAM)-TTTCCCGAAGCTCATCGCAGACTCC-(TAMRA)3′
**GAPDH**	forward 5′-ACGGGAAACCCATCACCAT-3′reverse 5′-CCAGCATCACCCCATTTGA-3′probe 5′(FAM)-TTCCAGGAGCGAGATCCCGTCAAG-TAMRA)3′

*TIMP-1, TIMP-2 and MMP-2 were detected by TaqMan realtime-PCR analysis.

### Protein detection by immunoblot

Frozen unfixed lung tissue was homogenized in lysis buffer as previously described [14]. Cultures cells were harvested using a cell scraper and lysed in the same buffer. Protein concentration was determined with a Bio-Rad DC protein assay (Bio-Rad, Hercules, CA). Lysates resolved on a 10% reducing SDS-PAGE gel were transferred to a nitrocellulose membrane. Blots were probed with the following antibodies: polyclonal rabbit-anti-rat-phospho Smad2 (Cell Signaling, Danvers, MA, # 3101, 1∶1000), monoclonal rabbit-anti-rat-phospho Smad3 (Cell Signaling, Danvers, MA, # 9520, 1∶1000), polyclonal rabbit-anti-rat-Smad2/3 (Cell Signaling, Danvers, MA, # 3102, 1∶1000), polyclonal rabbit-anti-rat-Smad4 (Cell Signaling, Danvers, MA, # 9515), polyclonal rabbit-anti-rat-Smad7 (R&D Systems, Wiesbaden, Germany, MAB2029, 1∶1000), polyclonal rabbit-anti-rat-cleaved Caspase-3 (Cell Signaling, Danvers, MA, # 9661, 1∶1000), polyclonal rabbit-anti-rat-Caspase-3 (Cell Signaling, Danvers, MA, # 9662, 1∶1000), polyclonal rabbit-anti-rat-poly (ADP-ribose) polymerase (PARP) (Cell Signaling, Danvers, MA, # 9542, 1∶2000), monoclonal mouse-anti-rat-proliferating cell nuclear antigen (PCNA) (DAKO, Glostrup, Denmark, Clone PC10, M0879, 1∶10.000). Monoclonal mouse-anti-rat-β-Actin (Cell Signaling, Danvers, MA, # 3700, 1∶1000) served as a loading control. Anti-mouse IgG, HRP-linked (Cell Signaling, Danvers, MA, # 7076, 1∶2000), and HRP-linked anti-rabbit IgG (Cell Signaling, Danvers, MA, # 7074, 1∶2000) were used as secondary antibodies.

Densitometric analysis of protein bands was performed using Advanced Image Data Analyzer-Software (Version 4.15, Fuji Photo Film Co., Omiyama, Japan) and Bio-Rad ImageLab software (Bio-Rad, Munich, Germany). Band intensities from samples were normalized for loading using the β-actin band from the same sample.

### Immunostaining of lung tissue sections

Expression of Smad molecules was assessed on 1-µm tissue sections, prepared as described above for morphometric analysis. Antigen retrieval and quenching of endogenous peroxidase activity with 3% (vol/vol) H_2_O for 20 min was performed. Sections were incubated with the relevant primary antibody: polyclonal rabbit-anti-rat-phospho Smad2 (Cell Signaling, Danvers, MA, # 3101, 1∶1000) or monoclonal rabbit-anti-rat-phospho Smad3 (Cell Signaling, Danvers, MA, # 9520, 1∶100). Immune complexes were visualized with an avidin/biotin-DAB (3,3′-diaminobenzidine) detection system (Vector Lab, Burlingame, CA, USA). Each slide was counterstained with hematoxylin.

### Optimization of the multiplicity of infection (M.O.I.)

NIH/3T3, MLE-12 and mouse endothelial cells were seeded in 24-well tissue culture plates at a density of 1×10^4^/well, incubated for approximately 12 h until 50% confluent, washed with PBS and then incubated in serum-free OptiMEM medium (Invitrogen, Darmstadt, Germany; 1000 µl per well) for 30 min at 37°C before addition of the virus. To define the best suitable multiplicity of infection (M.O.I.), an adenoviral LacZ vector (AdLacZ) carrying a β-galactosidase reporter gene was used. AdLacZ was diluted in PBS to a final concentration of 1×10^6^ plaque forming units (p.f.u.)/µl, applied to the cells with increasing M.O.I. (10, 50, 100, 200 and 500 µl per well), follwed by incubation of the cells at 37°C for 3 h. The volume of the culture medium was then increased by addition of OptiMEM (1500 ml per well). After incubation for 6 h the medium was replaced by the appropriate culture medium containing FCS and the incubation was continued for a total of 48 h. All experiments were performed in triplicate. After 48 h of incubation cells were fixed in 2% formaldehyde/0.2% glutaraldehyde for 15 min, washed with PBS supplemented with 0.02% Nonidet P40 and analyzed for nuclear bacterial β-galactosidase activity indicated by the characteristic blue staining in a PBS solution containing K_3_Fe(CN)_6_ (5 mM), K_4_Fe(CN)_6_ (5 mM), MgCl_2_ (2 mM), 0.02% Nonidet P40, 0.01% sodium deoxycholate and X-gal (5-bromo-4-chloro-3-indolyl-β-*D*-galactopyranoside dissolved in *N,N*-dimethylformamide) at a concentration of 0.8 mg/ml. The cells were stained overnight protected from light.

### Infection with an adenoviral Smad7 vector

The E1A/E1B-deleted adenoviral vector AdSmad7 was diluted in PBS to a final concentration of 1×10^6^ p.f.u./µl and applied to the cells (NIH/3T3, MLE-12, and mouse endothelial cells) at an M.O.I. of 100. The incubation was continued for a total of 48 h. All experiments were performed in triplicate.

AdSmad7-infected cells were stimulated with TGF-β1 (2 ng/ml) for 12 and 24 h. The cells were then lysed and processed for mRNA extraction. Uninfected cells served as control.

### Data analysis

The results of real-time RT-PCR were calculated based on the ΔΔCt method and expressed as fold induction of mRNA expression compared to the corresponding control group (1.0-fold induction). For quantitative immunoblot analysis densitometry was performed and values were normalized to β-actin. Two-tailed Mann-Whitney test and one-way ANOVA followed by a Bonferroni post-test were used to assess the significance of differences between IUGR and control animals at given time points. A *p* value<0.05 was considered as significant. All results are shown as means ± standard error of the mean.

## Results

### Auxometry of neonatal and adult rats after IUGR

**Figure 1:**
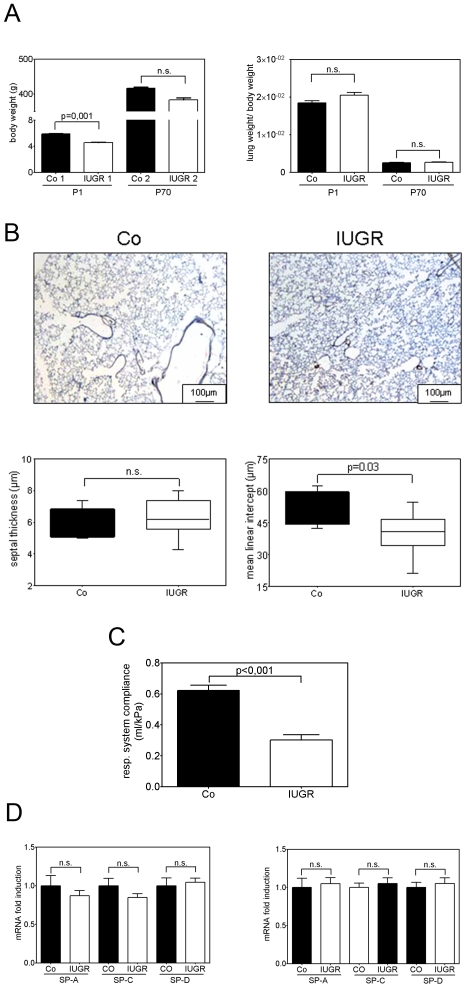
Body weight, respiration and physiological lung parameters of IUGR rats. A: Body weight (g) and relative lung weight (lung weight/body weight) of rats after IUGR (white bars) and control rats (black bars) at days P1 and P70; n = 8–15 for each bar. B: Architectural changes in lung structure were evident in hematoxylin and eosin-stained lung sections from IUGR rats and control rats at day P70. Measurement of septal thickness (µm) and mean linear intercept (MLI; µm) in IUGR rats and control rats (CO) at day P70; n = 6–10 for each bar. C: Assessment of respiratory system compliance by whole body plethysmography in IUGR rats (white bars) and in the control group (CO; black bars) at P70. n = 15–17 for each bar. D: Expression pattern of genes encoding surfactant protein A (SP-A), SP-C, and SPD. IUGR rats (white bars) and control group (black bars). n = 6–15 for each bar. The significance for each bar is indicated by p values, IUGR vs. Co, n.s. = not significant; two-tailed Mann-Whitney test.

A marked effect of low protein diet during gestation on growth, as assessed by body length and body weight, was observed (Figure 1A). At day P1 average body weight (5.86±0.092 g) of the undernourished pups (IUGR) was significantly lower than that of age-matched pups of mothers fed with normal protein (control group: 4.55±0.068 g). However, by P70 the IUGR group exhibited a slightly reduced body mass (383±6.32 g) in comparison with the control group (416±4.61 g). This difference was not significant when tested by one-way ANOVA followed by Bonferroni post-test (Figure 1A). Thus, low protein diet during gestation led to IUGR without affecting survival or adult body weight.

### Lung morphology of adult rats formerly affected by IUGR

Total lung weight at P1 and P70 did not differ between IUGR and control animals. However, alveolar development was impaired in IUGR animals, evident by fewer and larger air spaces (Figure 1B), compared to animals with appropriate birth weight. The mean linear intercept (MLI) is roughly inversely proportional to the alveolar surface (37). By P70, IUGR animals exhibited a MLI approximately 30% lower than that of the control group. To evaluate the interstitium and accumulation of ECM components we assessed septal thickness. There were no differences between IUGR and Co.

### Respiratory parameters of adult rats formerly affected by IUGR

A marked effect of IUGR on dynamic respiratory compliance (C_dyn_) was observed at P70. IUGR animals exhibited a C_dyn_ 40% lower than that of age-matched controls (Figure 1C). C_dyn_ reflects the elasticity and function of the pulmonary connective tissue. Interestingly, lung compliance was significantly lower in IUGR animals under baseline conditions.

### Expression pattern of surfactant protein A (SP-A), SP-C, SP-D

To address surface tension as a regulator of lung compliance, we assessed mRNA expression of genes encoding surfactant protein A (SP-A), SP-C, and SP-D at P1 and P70. There was no significant difference detectable, neither at P1 nor at P70 (Figure 1D).

### Effect of IUGR on TGF-β-induced ECM proteins and modulators of the ECM in neonatal and adult rat lungs

**Figure 2:**
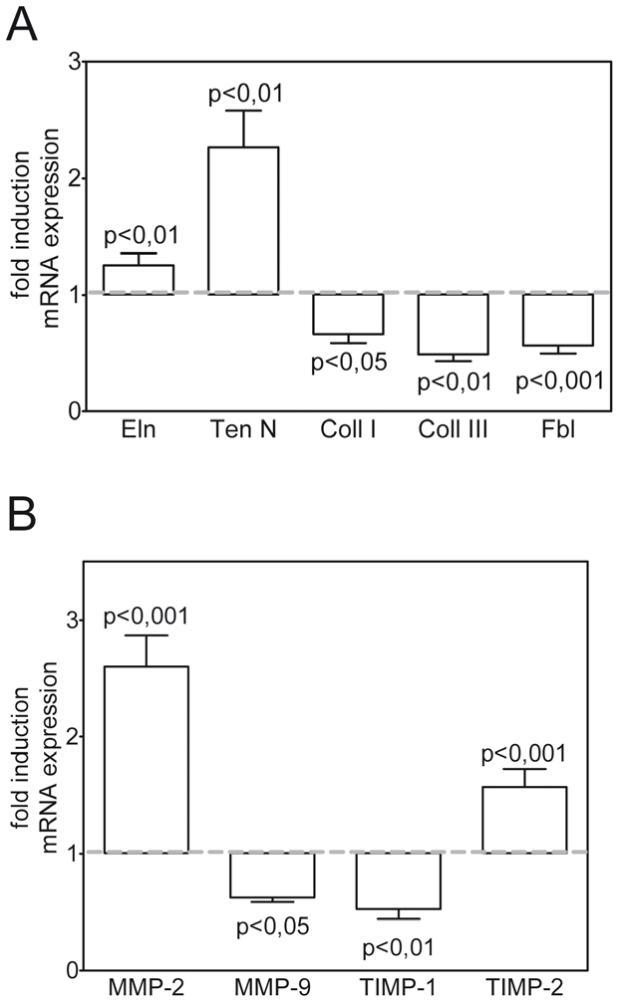
Effect of IUGR on the expression of extracellular matrix (ECM) proteins and modulators of the ECM in neonatal rat lungs. Expression of TGF-β-regulated genes encoding elastin (Eln), tenascin N (Ten), collagens (Coll) I, Coll III, and Fibrillin (A), and genes of ECM modulators including matrix metalloproteinase (MMP)-2, MMP-9, tissue inhibitor of metalloproteinases (TIMP)-1, and TIMP-2 (B) in lungs extracted at day P1 from neonatal rats after IUGR and control rats. The mRNA expression, illustrated as relative fold induction, was assessed by real-time PCR. The control group was normalized to 1 as indicated by a scattered line; n = 15 for each group. The significance for each bar is indicated by p values, IUGR vs. Co; two-tailed Mann-Whitney test.

To address whether the dramatic alterations in dynamic respiratory compliance (C_dyn_) and respiratory system resistance (Res) in IUGR animals are associated with altered expression of ECM components, we assessed the mRNA levels of genes encoding ECM molecules. Expression of collagen I, collagen III, fibrillin (Figure 2A) and the ECM regulators MMP-9 and TIMP-1 (Figure 2B) was downregulated at the critical developmental time point P1. In contrast, the expression of elastin (Eln), tenascin N (TenN) (Figure 2A), as well as that of MMP-2, a regulator of ECM remodeling enzymes, and its inhibitor TIMP-2 (Figure 2B) was elevated. These results indicate a dysregulated composition and remodelling of the ECM during the stage of alveolarization.

### Effect of IUGR on the expression of TGF-β1 and the methylation of CpG islands of the promoter region of TGF- β1

**Figure 3:**
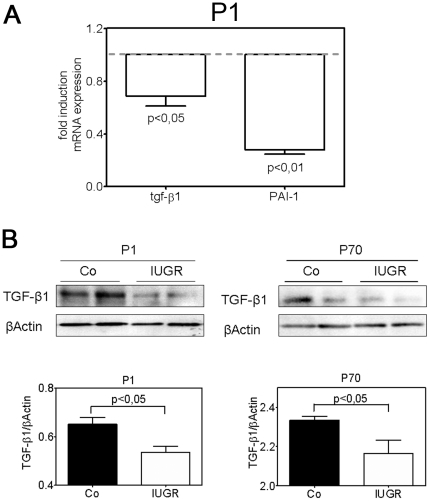
Effect of IUGR on the expression of TGF-β1 in lungs of neonatal and adult rats. A: Expression of genes encoding TGF-β1 and TGF-β-inducible plasminogen activator inhibitor-1 (PAI-1) during late lung development (day P1). The mRNA expression was assessed by quantitative real-time PCR. The control group was normalized to 1 as indicated by a scattered line; n = 15 for each group. The significance for each bar is indicated by p values, IUGR vs. Co; two-tailed Mann-Whitney test. B: representative immunoblots illustrating expression of TGF-β1 in lungs extracted at day P1 and P70 from rats with and without IUGR. β-actin served as loading control. Immunoblot data were quantified for both day P1 and P70; n = 6 for each bar. The significance for each bar is indicated by p values, IUGR vs. Co; two-tailed Mann-Whitney test.

To address whether the alterations in the expression of ECM components at the critical phase of lung development at P1 are associated with an altered expression of the growth factor TGF-β1 in the neonatal lungs of IUGR rats, we measured the expression of the gene encoding TGF-β1 and the TGF-β responsive gene PAI-1. Both TGF-β1 mRNA and protein were decreased in IUGR pups at P1 (Figure 3A, Figure 3B), consistent with the downregulation of expression of the TGF-β-responsive gene PAI-1 at P1 (Figure 3A). TGF-β1 protein was detected at lower amounts in lungs of IUGR animals at P70 (Figure 3B, 3C).

Next, we wanted to investigate why the expression of the ligand TGF-β1 is changed after IUGR. Therefore we analyzed the methylation of CpG islands in the promoter region of the TGF-β1 gene by PCR amplification of bisulfite-treated DNA, separation by agarose gel electrophoresis, and gel extraction and purification of the PCR products. Analysis of the PCR products did not reveal any significant difference of methylation in lungs of IUGR rats compared to the controls (data not shown).

### Effect of IUGR on the expression of TGF-β signaling molecules in rat lungs

**Figure 4:**
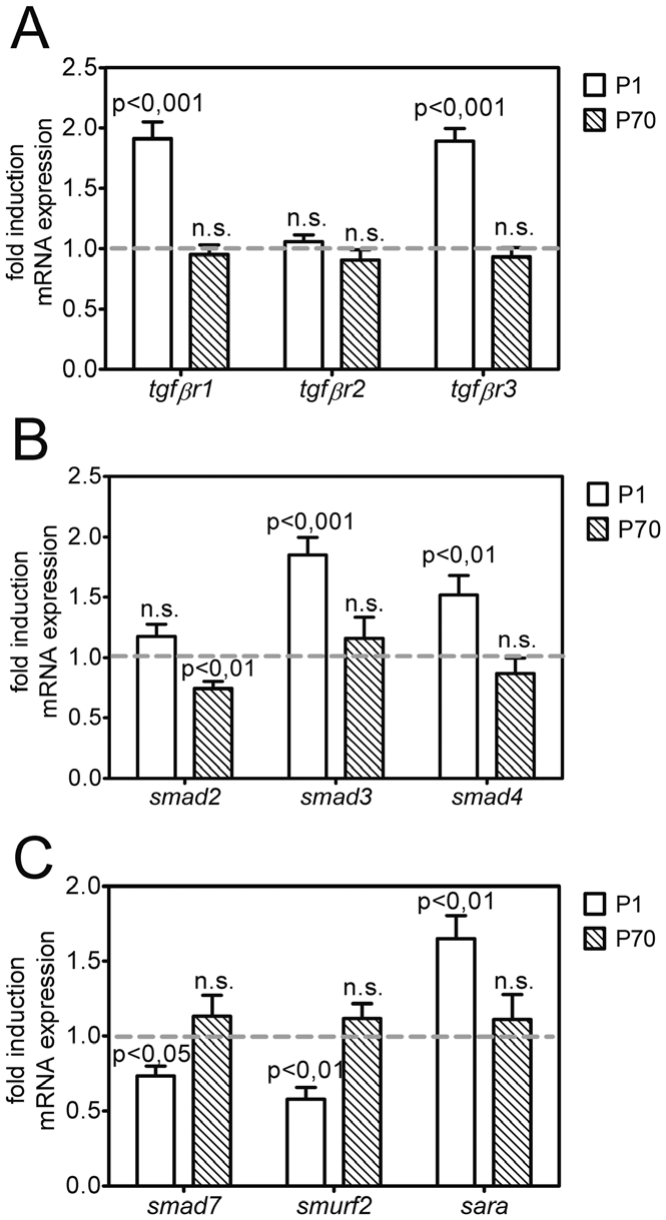
Expression of the TGF-β signaling machinery in lungs of neonatal and adult rats. Changes in the expression of genes encoding components of the TGF-β signaling machinery as assessed by real-time RT-PCR at days P1 and P70; n = 8–15 for each bar. The significance for each bar is indicated by p values, IUGR vs. CO; two-tailed Mann-Whitney test. A: Expression of genes encoding the TGF-β receptors *tgfbr1*, *tgfbr2* and *tgfbr3* at P1 (white bar) and P70 (striped bar). B: Expression of genes encoding the regulatory *smad2*, *smad3* and *smad4* at days P1 (white bar) and P70 (striped bar). C: Expression of genes encoding the inhibitory *smad7*, *smurf2* and smad anchor for receptor activation (*sara*) at days P1 (white bar) and P70 (striped bar).

Next, we assessed expression of the components of TGF-β signaling by quantitative real-time PCR. The results demonstrate a significant increase of the transforming growth factor receptor type I (TβRI) and TβRIII in lungs of IUGR animals, but no changes for TβRII at P1. At P70 the expression of the receptors did not differ between the two groups (Figure 4A). The mRNA expression of the regulatory Smad2 was reduced, whereas the mRNA levels of Smad3 and Smad4 were significantly increased at P1. At P70 Smad2 expression was downregulated, while no remarkable difference for Smad3 or Smad4 was observed (Figure 4B). Expression of the inhibitory molecule of the TGF-β system, Smad7, was slightly decreased and expression of Smad-specific E3 ubiquitin protein ligase 2 (Smurf2), which inhibits Smad2 and Smad3, was significantly reduced. The expression of Smad anchor for receptor activation (Sara) as a protein presenting Smad2 and Smad3 to the TGF-β-receptors, was significantly increased (Figure 4C).

**Figure 5:**
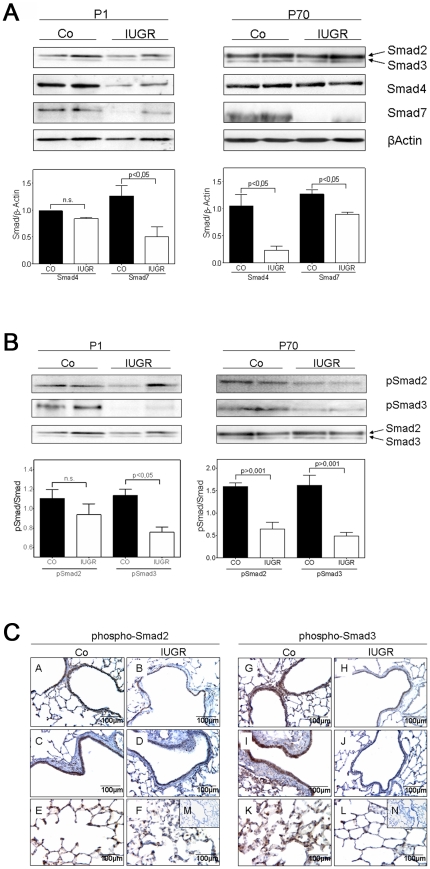
IUGR alters expression and phosphorylation of Smad proteins in rats. A: Representative immunoblots illustrating the expression of TGF-β-specific Smad2, Smad3, the co-Smad, Smad4, and the inhibitory Smad, Smad7, in lungs extracted at days P1 and P70 from rats with and without IUGR. β-actin served as loading control. Immunoblot data were quantified for Smad4 and Smad7 for both days P1 and P70 (Co as black bar, and IUGR as white bar); n = 4–6 for each bar. The significance for each bar is indicated by p values, IUGR vs. CO; two-tailed Mann-Whitney test. B: The expression of active TGF-β signaling components in lung homogenates of rats with and without IUGR was analyzed by immunoblotting of phosphorylated (p) and total Smad2 and Smad3. β-actin served as loading control. Immunoblot data were quantified for pSmad2 and pSmad3 for both days P1 and P70 (Co as black bar, and IUGR as white bar); n = 4–6 for each bar. The significance for each bar is indicated by p values, IUGR vs. CO; two-tailed Mann-Whitney test. C: Immunhistochemical localization and expression pattern of pSmad2 and pSmad3 in lungs of rats with IUGR (right column) and without IUGR (left column). A–F: representative fields illustrating the expression and localization of pSmad2 in bronchi (A–D) and in the alveoli (E–F) of lungs extracted on day P70. G–L: representative fields illustrating the expression and localization of pSmad3 in bronchi (G–J) and in the alveoli (K–L) of lungs extracted on day P70. M–N: negative control.

Additionally, where antibodies were available, lung homogenates at P1 and P70 were probed by immunoblotting to investigate whether IUGR due to undernourishment during gestation resulted in changes on the protein level between IUGR and control groups. Indeed, pronounced alterations were observed for some intracellular signaling components of the TGF-β system. The abundance of co-Smad4 and of inhibitory Smad7 was decreased at P1 (Figure 5A). At P70 the expression of Smad4 was unchanged, while expression of Smad7 was persistently downregulated (Figure 5A). The expression of regulatory Smad2 and Smad3, transducers of TGF-β signals, was altered neither at P1 nor at P70 (Figure 5A).

### Effect of IUGR on the activity and localization of TGF-β signaling in rat lungs

The results obtained so far indicate a regulation of the expression of the TGF-β system due to IUGR. To further elucidate whether the activity of TGF-β signaling was changed in lungs of IUGR rats, we analyzed the phosphorylation of intracellular Smad2 and Smad3 by immunoblot. At P1 and P70 phosphorylation of Smad2 and Smad3 was significantly diminished (Figure 5B), indicating that the activity of TGF-β signaling was decreased in lungs of IUGR rats.

To localize the activity of TGF-β signaling within the different compartments of the lung we performed immunohistochemical analysis of lung tissue at P70. Consistent with the immunoblotting data, the phosphorylation of both Smad2 and Smad3 was less in the bronchi and in the alveoli of lungs after IUGR (Figure 5C).

### Effect of IUGR on apoptosis in neonatal and adult rat lungs

**Figure 6:**
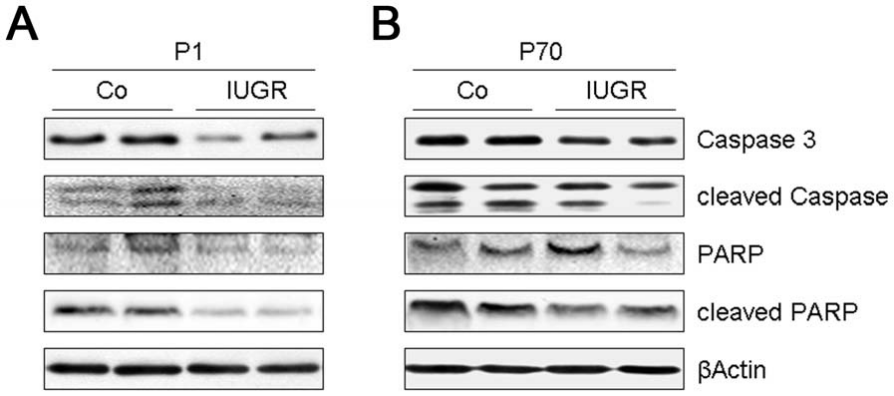
Effect of IUGR on apoptosis in lungs of rats at days P1 and P70. Apoptosis is assessed by cleaved caspase-3 and cleaved fragment of Poly (ADP-ribose) polymerase (PARP). A: Representative immunoblots illustrating the expression of cleaved and total caspase-3, fragments of PARP and total PARP in lung homogenates of rats with IUGR and without IUGR (Co) at *day* P1 (A) and P70 (B). The β-actin served as loading control; n = 4–6 for each bar.

The observed impaired pulmonary TGF-β signaling in IUGR rats is likely to influence apoptosis and proliferation during the vulnerable perinatal period and may thereby contribute to the increase in alveolar surface and lung tissue in adulthood. Therefore, we next sought to examine key markers of apoptosis and proliferation in the developing lung at P1 and in the adult lung at P70. We assessed expression of caspase-3 and polyclonal rabbit-anti-rat-poly (ADP-ribose) polymerase (PARP) by immunoblotting. Total caspase 3 levels were reduced in lungs of IUGR rats at P1 at P70, whereas PCNA levels were not changed. Additionally, cleaved caspase-3 and the cleaved fragment of PARP were markedly diminished after IUGR at both time points, indicating decreased apoptosis in lungs after of IUGR rats (Figure 6).

### Effect of the inhibition of the TGF-β signaling by adenoviral Smad7 on the expression of extracellular matrix proteins

**Figure 7:**
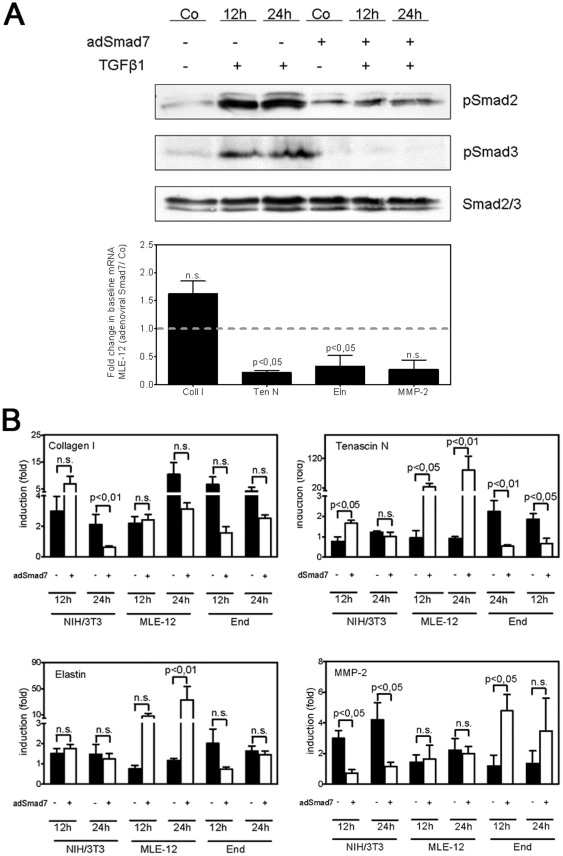
Effect of the inhibition of intracellular TGF-β signaling by adenoviral Smad7 overexpression on the expression of ECM components *in vitro*. A: Basal expression of components of the TGF-β signaling pathway in MLE-12 cells following infection with adenoviral Smad7 (AdSmad7) and stimulation with TGF-β1 (2 ng/ml) for either 12 h or 24 h. Representative immunoblots illustrating the expression and phosphorylation of Smad2 and Smad3 in MLE-12 cells are shown. Changes in the basal mRNA levels of genes encoding collagen I (ColI), tenascin N (TenN), elastin (Eln) and metallo-matrixproteinase-2 (MMP-2) in MLE-12 cells infected by AdSmad7. Data represent the mean relative fold change in mRNA expression assessed by real-time RT-PCR in MLE-12 cells 24 h after infection with AdSmad7. B: Induction of these four genes in native fibroblasts (NIH/3T3), alveolar cells (MLE-12) and endothelial cells (End) (black bars) and after infection with AdSmad7 (white bars) one day prior to a stimulation with TGF-β1 (2 ng/ml) for 12 h or 24 h. Fold changes were calculated by: (ΔΔC_t adSmad7, stim_/ΔΔC_t adSmad7, unstim_), and (ΔΔC_t control, stim_/ΔΔC_t control, unstim_), where adSmad7 denotes infection with AdSmad7; control, no infection with AdSmad7; stim, ligand-stimulated; unstim, unstimulated. The significance for each bar is indicated by p values, AdSmad7 vs. control.

The inhibition of TGF-β signaling by AdSmad7 was confirmed by immunoblotting of both phospho-Smad2 and phospho-Smad3. Additionally we assessed baseline transcriptional activity of the cells infected by AdSmad7. Accordingly, the induction of transcription was normalized to AdSmad7/Co (Figure 7A).

To assess the impact of TGF-β signaling on the expression of ECM components, we next performed cell culture experiments. Phosphorylation of Smad2 and Smad3 was inhibited by infecting NIH/3T3 cells (fibroblasts), MLE-12 epithelial cells and murine endothelial (mEnd) cells with a constitutively-expressing Smad7 adenoviral vector (AdSmad7). Inhibition of intracellular TGF-β signaling led to cell type-dependent expression. Collagen I was significantly downregulated in NIH/3T3 cells and slightly, but not significantly in MLE-12 and mEnd cells. Messenger RNA expression of tenascin N was slightly increased in NIH3T3 and dramatically upregulated in MLE-12, whereas no changes could be detected in mEnd. The expression of elastin increased strikingly in MLE-12 cells after inhibition of the Smad-dependent pathway. Matrix metalloproteases-2 (MMP-2) expression was not altered in MLE-12 cells, but was down- or upregulated in NIH3T3 and mEnd cells, respectively (Figure 7B). Taken together, these data demonstrate a direct effect of the inhibition of TGF-β signaling by AdSmad7 on ECM components. The results are consistent with our *in vivo* data and underline the impact of a decreased TGF-β activity on the ECM.

## Discussion

Several studies have examined ECM molecules and morphometric parameters of lungs after IUGR [38], but none so far has addressed lung function and the impact of growth factors such as TGF-β on the pulmonary system. Here, we aimed at elucidating mechanistic clues linking IUGR and subsequent changes in the pulmonary architecture.

Dynamic lung compliance is a dimension for the lung scaffold and ECM, and the pulmonary ability to distend in response to pressure. Our data demonstrate that IUGR following maternal isocaloric protein restriction during gestation leads to a reduced dynamic compliance. This is in line with studies demonstrating an airway disease in former IUGR infants [12]. Further, morphometric analysis at P70 revealed a decreased MLI after IUGR, indicating an increase of alveolar tissue, whereas there was no alteration of septal thickening. These results are in contrast to previously reported morphometric lung analyses subsequent to IUGR demonstrating an impaired alveolarization [16], [17]. However, those previous studies differ from our in various points: (1) species, (2) induction of IUGR and (3) catch up growth. Consistent with our observations, expression of both ECM components (elastin, tenascin N, collagens I and III) and ECM regulators (MMP-2 and TIMP-2) was markedly dysregulated. This is in contrast to the findings of Maritz et al. [38], who reported a lower number of alveoli per respiratory unit and thicker interalveolar septa in IUGR lambs. In addition, we investigated the surfactant proteins, based on the fact that lung compliance is determined by them, but did not detect any difference. The cause of IUGR is of utmost importance and determines the phenotype later in life [39]. Maritz *et al.* used a model of placental insufficiency, whereas the present study is based on a low protein diet model. That may explain, at least in part, the different observations.

How could IUGR dysregulate ECM components during lung development? The process of alveolarization is regulated by growth factor-mediated interaction of different cell types [18], [19]. The TGF-β family controls cell proliferation, transformation and apoptosis, as well as ECM deposition and remodelling [8], [18], [19]. There are studies demonstrating that several modificators, such as RAGE-products [40] and hypoxia [41], are involved in lung disease and regulation of TGF-β signaling. Recent studies of our group did not indicate placental or fetal hypoxia subsequent to IUGR induced by low protein diet during gestation. Overexpression of TGF-β1 during gestation leads to septal thickening and lung fibrosis [31], [42], whereas Smad3-deficient mice develop a hypoplastic lung [32], [43]. Here we show, for the first time, that IUGR in rats decreases pulmonary TGF-β signaling persistently. TGF-β1 mRNA and protein levels were reduced immediately after birth and at P70. Such persistent changes could be the result of epigenetic modifications, for example methylation of promoter regions (CpG islands) influencing gene expression and possibly inducing gene silencing. Some studies revealed an effect of IUGR due to maternal protein restriction on DNA methylation and on the expression of essential growth or transcription factors [44], [45], [46]. We could not detect differences between IUGR and control animals. However, other epigenetic mechanisms of transcriptional regulation via transcription factors may contribute to the altered TGF-β1 expression. In line with this, the phosphorylation of both Smad2 and Smad3 is significantly derogated at P1 and P70 in the rat lung. TGF-β regulates the expression and secretion of some ECM molecules, including collagens, fibrillin, and matrix-metabolizing enzymes [7], [8], [47]: the MMPs and their cognate inhibitors, TIMPs. During early and late lung development both ECM components and MMP-2/TIMP-1 are strongly expressed in humans [48] and mice [49], and their deposition in the ECM and its remodeling plays a pivotal role in alveolarization. In our study, we have illustrated that the levels of collagen I, collagen III, and fibrillin mRNA are consistently decreased. In animal models of arrest of alveolarization, MMP-2 is reduced and TIMP-1 is elevated [50], [51]. In contrast to these results, we demonstrate an increased alveolarization and alveolar mass after IUGR, and opposing regulation of TIMP-1 and MMP-2. Additionally, we show that inhibition of the TGF-β activity in fibroblasts by adenoviral Smad7 leads to reduced mRNA expression of TGF-β-regulated ECM molecules. However, it is essential to differentiate between the cell types of the compartments of the lung: in epithelial cells (MLE-12) inhibition of TGF-β signaling led to a tremendous upregulation of elastin and tenascin N, whereas elastin expression was unaffected in fibroblasts (NIH/3T3) and murine endothelial cells, and tenascin N expression was even decreased in murine endothelial cells. Taken together, our data *in vivo* and *in vitro* suggest that an abnormal downregulation of pulmonary TGF-β activity after IUGR has an impact on the composition and function of the ECM contributing to an impaired lung function.

**Figure 8:**
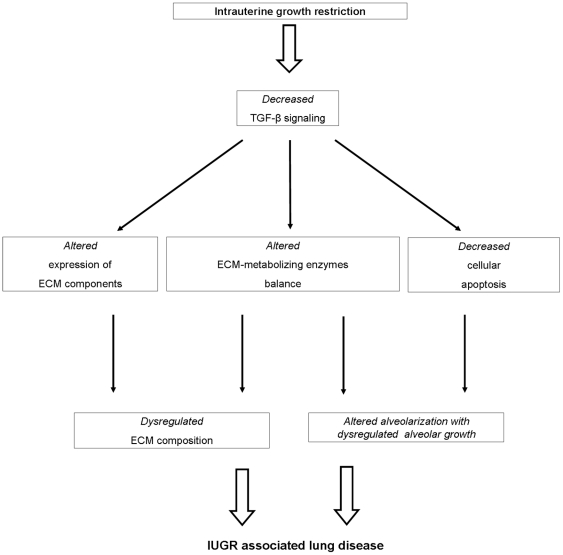
The role of transforming growth factor (TGF)-β signaling in lung disease subsequent to intrauterine growth restriction (IUGR). A proposed model depicting the effects of decreased TGF-β signaling during the development of IUGR-associated lung disease is shown. ECM - extracellular matrix.

What other role may the TGF-β system play during lung development in IUGR animals? Proliferation and differentiation of type II pneumocytes are key steps in the process of alveolarization and regulated by TGF-β [52], [53]. Our study shows that IUGR may result in a decreased activation of the TGF-β system accompanied by diminished expression and cleavage of caspase-3 and reduced cleavage of PARP, indicating clearly disturbed apoptotic processes at both time points investigated. These findings are endorsed by the fact that caspase-3 is a downstream molecule of the TGF-β system [54], whereby TGF-β signaling has potent antiproliferative and pro-apoptotic effects on epithelial cells [55], [56], [57], [58]. Moreover, we demonstrate that tgfbr3 expression is significantly upregulated in IUGR lungs at P1. Consistent with these results, another group postulates that TGF-β receptor III (TβRIII) may act as a protective factor in apoptotic processes in cardiac fibroblasts by negative regulation and inhibition of TGF-β signaling [59]. Considering these data, it is conceivable that diminished TGF-β signaling in lungs after IUGR inhibits apoptosis in fibroblasts and alveolar epithelial cells, thereby contributing to an abnormal growth of pulmonary tissue (Figure 8).

Additionally, compensatory mechanisms occur in order to counter-regulate the reduced phosphorylation of Smad2/3 by downregulation of the inhibitory intracellular molecules Smad7 and Smurf2. Furthermore, phosphorylation of Smad2/3 supporting molecules, e.g. Sara, is upregulated indicating a compensation for the reduced activity of the TGF-β system.

There two major reasons for IUGR: first, placental insufficiency with a combination of hypoxemia, inflammatory reaction and nutrient restriction, second, maternal undernourishment due to low protein diet. Of the different animal models of IUGR, we chose the low protein diet model in the rat, based on the fact that it is characterized by a low birth weight, development of arterial hypertension and pronounced responsiveness to inflammatory processes. However, undernourishment is not the leading cause of IUGR in the western world, but in the developing countries. Hence, the data presented in our study may be limited to a certain group of IUGR infants and not completely alienable to IUGR induced by placental insufficiency. Furthermore, rat lungs at birth are at an earlier developmental stage than lungs of human neonates born at term and therefore comparable to preterm infants.

Taken together, the data presented here suggest that IUGR affects lung development and lung function by at least two functional consequences: *1)* IUGR attenuates TGF-β signaling after IUGR which leads to a dysregulated expression of ECM and ECM-remodeling components, and *2)* IUGR decreases apoptosis in the lung. This significantly contributes to the altered lung development and impaired lung function seen after IUGR.
